# Biochar and slow-releasing nitrogen fertilizers improved growth, nitrogen use, yield, and fiber quality of cotton under arid climatic conditions

**DOI:** 10.1007/s11356-021-16576-6

**Published:** 2021-10-01

**Authors:** Sobia Manzoor, Muhammad Habib-ur-Rahman, Ghulam Haider, Iqra Ghafoor, Saeed Ahmad, Muhammad Afzal, Fahim Nawaz, Rashid Iqbal, Mubashra Yasin, Tanveer-ul-Haq, Subhan Danish, Abdul Ghaffar

**Affiliations:** 1 Department of Agronomy, Muhammad Nawaz Shareef University of Agriculture, Multan, Pakistan; 2 Crop Science Group, Institute of Crop Science and Resource Conservation (INRES), University Bonn, Bonn, Germany; 3 Department of Plant Biotechnology, Atta-ur-Rahman School of Applied Biosciences, NUST, Islamabad, Pakistan; 4 Legume Research Unit, Molecular Biology Lab, Department of Plant Production, King Saud University, Riyadh, Saudi Arabia; 5 Department of Nutritional Crop Physiology, Institute of Crop Science (340 h), University of Hohenheim, 70599 Stuttgart, Germany; 6 Department of Agronomy, Faculty of Agriculture and Environment, The Islamia University of Bahawalpur, Bahawalpur, Pakistan; 7 Sugarcane Research Institute, Ayub Agricultural Research Institute, Faisalabad, Pakistan; 8 Department of Soil Science, MNS-University of Agriculture Multan, Multan, Pakistan; 9 Department of Soil Science, Faculty of Agricultural Sciences and Technology, Bahauddin Zakariya University, Multan, Punjab 60800 Pakistan

**Keywords:** Photosynthetic and transpiration rate, Stomatal conductance, Partial factor productivity, NUE, Fiber quality

## Abstract

The efficiency of nitrogenous fertilizers in South Asia is on a declining trajectory due to increased losses. Biochar (BC) and slow-releasing nitrogen fertilizers (SRNF) have been found to improve nitrogen use efficiency (NUE) in certain cases. However, field-scale studies to explore the potential of BC and SRNF in south Asian arid climate are lacking. Here we conducted a field experiment in the arid environment to demonstrate the response of BC and SRNF on cotton growth and yield quality. The treatments were comprised of two factors, (A) nitrogen sources, (i) simple urea, (ii)neem-coated urea, (iii)sulfur-coated urea, (iv) bacterial coated urea, and cotton stalks biochar impregnated with simple urea, and (B) nitrogen application rates, N_1_=160 kg ha^-1^, N_2_ = 120 kg ha^-1^, and N_3_ = 80 kg ha^-1^. Different SRNF differentially affected cotton growth, morphological and physiological attributes, and seed cotton yield (SCY). The bacterial coated urea at the highest rate of N application (160 kg ha^-1^) resulted in a higher net leaf photosynthetic rate (32.8 μmol m^-2^ s^-1^), leaf transpiration rate (8.10 mmol s^-1^), and stomatal conductance (0.502 mol m^-2^ s^-1^), while leaf area index (LAI), crop growth rate (CGR), and seed cotton yield (4513 kg ha^-1^) were increased by bacterial coated urea at 120 kg ha^-1^ than simple urea. However, low rate N application (80 kg ha^-1^) of bacterial coated urea showed higher nitrogen use efficiency (39.6 kg SCY kg^-1^ N). The fiber quality (fiber length, fiber strength, ginning outturn, fiber index, and seed index) was also increased with the high N application rates than N2 and N3 application. To summarize, the bacterial coated urea with recommended N (160 kg ha^-1^) and 75% of recommended N application (120 kg ha^-1^) may be recommended for farmers in the arid climatic conditions of Punjab to enhance the seed cotton yield, thereby reducing nitrogen losses.

## Introduction

Cotton is the major fiber crop widely cultivated across the globe (Ali et al. 2015; Abbas, 2020). Pakistan is the fourth-largest cotton-producing country and the third-largest consumer of cotton products (Abbas, 2020). In Pakistan, cotton productivity is showing a declining trend over the last 5 years, mainly due to lower resource use efficiency, imbalanced fertilizer application, and especially reduced N use efficiency (NUE)(Rahman et al. 2016, 2018, 2019, 2020; Saleem et al. 2016). Simple urea fertilizer is widely used as an N fertilizer at three critical growth phases of a cotton crop (Yang et al. 2011). However, much N applied into the soil is lost to the environment (denitrification, nitrate leaching, and ammonia volatilization) which leads to soil degradation, emission of greenhouse gases, groundwater pollution, and ultimately reduced N use efficiency (Spiertz, 2009; Zhang et al. 2010). Hence, it is inevitable to improve the N use efficiency in cotton production systems of south Asia (Khan et al. 2017; Rahman et al. 2021). It is estimated that in a cotton production system, cotton can use only 33% of applied nitrogen during a season, while leaving 25% in the soil and 42% being lost to the environment (Tang et al. 2012). Advanced agronomic practices under different soil types can contribute to low NUE in the cotton production system (Nouri et al. 2020). For instance, the time of nitrogen application and the amount of applied nitrogen adversely affect the nitrogen use efficiency. Since farmers apply nitrogen at the time of sowing, it can be lost through leaching, volatilization, denitrification, immobilization, and clay fixation (Scheer et al. 2008). Secondly, cotton genotypes, cropping systems, and soil types may also contribute to low nitrogen use efficiency (Spiertz, 2009; Bronson, 2008; Nangial et al. 2019). Different measures such as the use of bio-inoculants(Khaitov et al., 2019), maintenance of optimum plant population and varying N application rates (Li et al. 2017), and the use of overhead sprinkler system (Bronson et al. 2011) have been shown to improve NUE in a cotton production system. In Pakistan, some measures have already been taken to improve NUE, including balanced use of fertilizers (Raza et al. 2018), adopting zero tillage (Khan et al. 2018), and use of slow-release fertilizers (Naz and Sulaiman, 2016).

Biochar has been advocated for a sustainable increase in soil organic matter (Ngo et al. 2014) and availability of essential plant nutrients (C, N, Ca, Mg, K, and P) on soil incorporation (Deluca et al. 2015). Soil incorporation of biochar can reduce the N losses and improve the N uptake (Zhang et al. 2010; Haider et al. 2015). It is used to reduce the N loss which is attributed to its more water holding ability and ions exchange capacity capture in macro-/meso-pores and adsorption through functional groups (Basso et al. 2013; Laird et al. 2010; Haider et al. 2016, 2017). Various studies have reported that soil incorporation of biochar decreases N loss through leaching and volatilization which is attributed to its water holding capacity (Knowles et al., 2011; Kameyama et al. 2012; Tian et al. 2017). It increases the microbial N cycling in the soil (Cayuela et al., 2014). Similarly, soil incorporation of biochar has improved the N use efficiency and leads to higher production of cotton and Quinoa (Yu et al. 2018; Haider et al. 2020). Furthermore, biochar reduces NH_3_ loss and increases N uptake and resulting in higher NUE in cotton production (Li, 2016).

SRNF are considered as a sustainable method of minimizing fertilizers losses by applying technically advanced methods of supplying nutrients to the crops in comparison to conventional fertilizers (Geng et al. 2015; Ghafoor et al. 2021). SRNF can reduce environmental pollution, improve the NUE, and can be a time-saving option (Jat et al. 2012; Kiran et al. 2010). However, there are limited studies available on SRNF and urea impregnated biochar to improve cotton growth, physiology, yield, quality, and N use efficiency. Thus, it is necessary to evaluate the response of SRNF and biochar for the improvement of N use efficiency in the cotton production system under arid environmental conditions. The major objective of the study was to explore if the SRNF and urea impregnated BC can improve N supply and thereby cotton yield? If yes, then at what level of N application can we achieve better quality cotton under an arid environment?

## Materials and methods

### Experimental site and environmental conditions

**Fig. 1:**
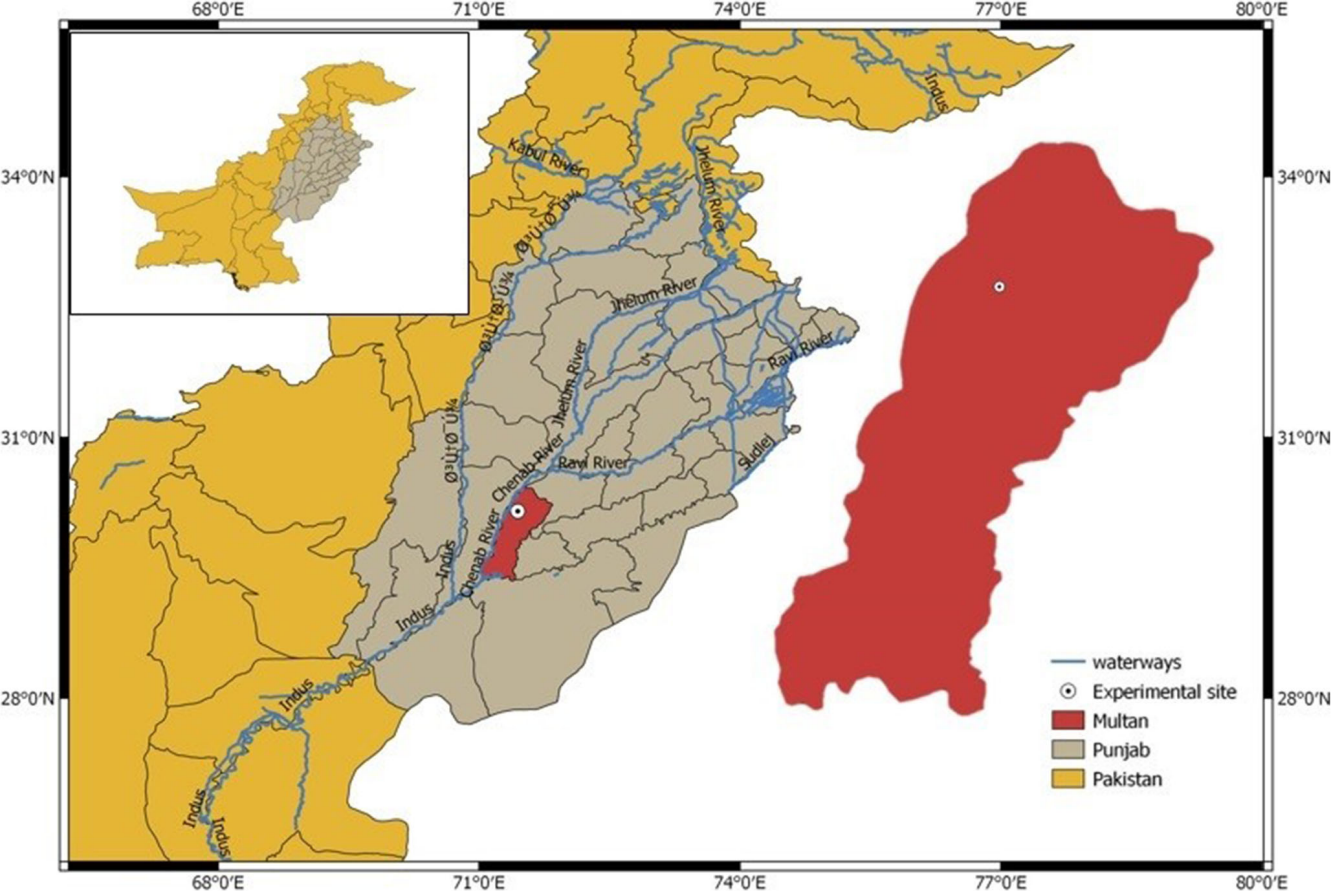
Location of the study area in arid climatic conditions of district Multan-South Punjab, Pakistan

**Figure 2:**
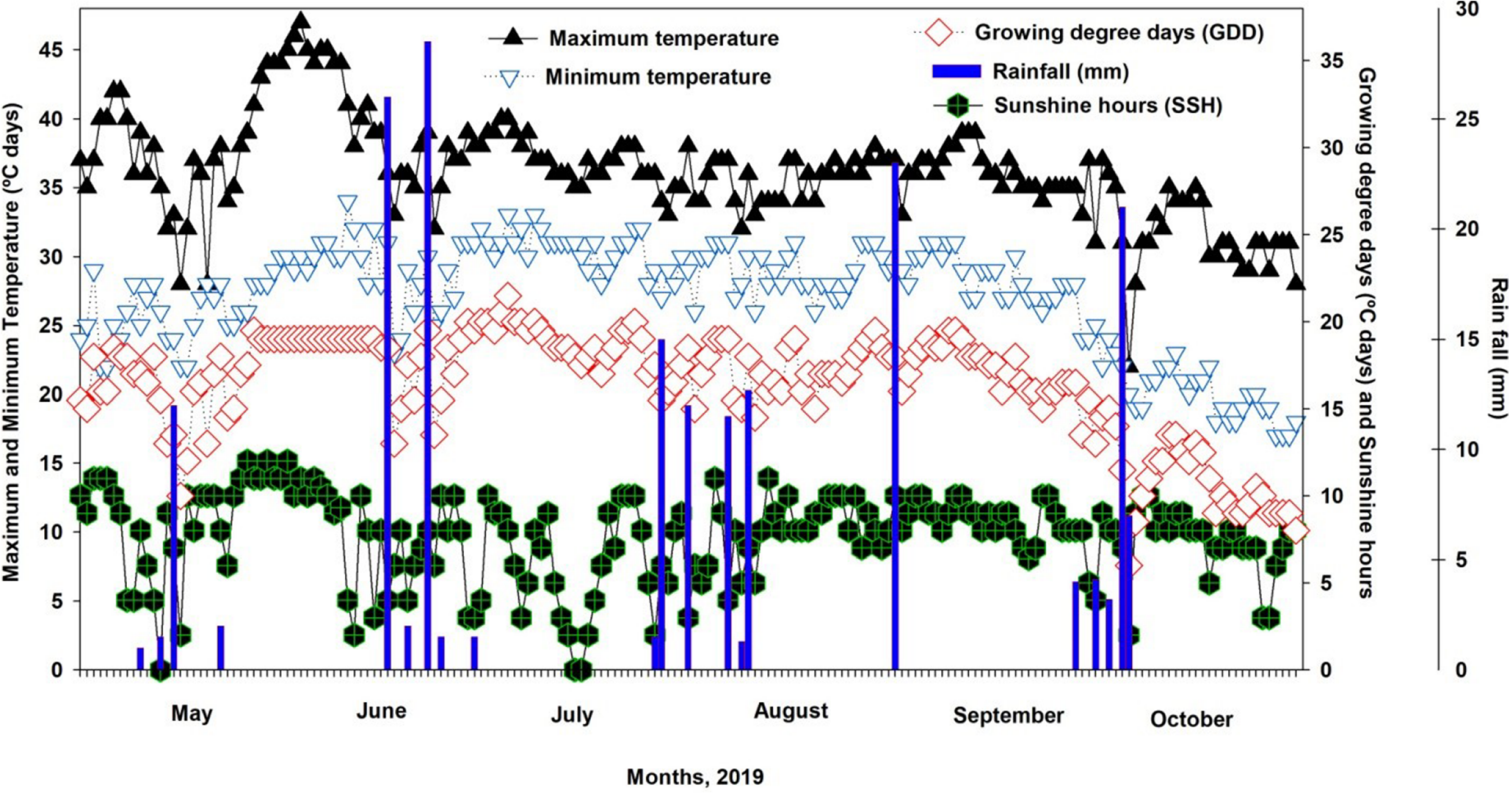
Weather variables (maximum, and minimum temperature, sunshine hours, growing degree days, and rainfall) during the cotton crop growing season in 2019

The field experiment was conducted under arid environmental conditions of South Punjab (30°15 N, 71°52 E), Pakistan, during the summer season 2019 (Figure 1). The elevation of the experimental site is about 178 m above sea level. The experimental site is considered as irrigated (canal irrigation for six months in a year). The environment is characterized by hot and moderately humid summer (March–June) and a warm and humid rainy season (July–September). Meteorological data collected from an automatic weather station of MNS-University of Agriculture, Multan, Pakistan, are presented in Figure 2. The meteorological unit (AWS) is located about 400 m from the experimental area. Before the experiment commenced, the soil samples were collected to depths of 0–30, 30–45, 45–60, and 60–90 cm from each experimental unit/plot and analyzed to assess the different soil properties in profile (Table 1). The soil was characterized as a loam textured soil.

**Table 1:** Physiochemical properties of soil profile before initiating the experiment

**Soil indicator**	**Soil depth (cm)**			
	**0–15**	**15–30**	**30–60**	**60–90**
Soil pH	8.00	8.00	8.00	8.00
Electrical conductivity (dS m^-1^)	3.88	3.90	3.90	3.92
Soil organic matter (%)	0.80	0.79	0.77	0.75
Soil available phosphorus (mg kg^-1^)	8.20	8.15	8.15	8.10
Soil available potassium (mg kg^-1^)	220	220	220	220
Soil available N (mg kg^-1^)	82.2	76.5	70.6	57.5

### Experimental details

#### Design and treatment details

The experiment was laid out in a randomized complete block design (RCBD) under a factorial arrangement with four replications. The treatments consisted of five slow-release N fertilizers (simple urea, NCU, SCU, bacterial (*Thiobacillus* oxidizing bacteria) coated urea and loaded biochar) and three N rates [100% of recommended (N_1_) =160 kg ha^-1^, 75% of recommended (N_2_) =120 kg ha^-1^ and 50%of recommended (N_3_) =80 kg ha^-1^). Each plot measured 3 m × 8 m and experimental plots were separated by 1-m distance to reduce the edge effects. The total number of experimental plots was *N* = 60.

#### Urea-coated products and fertilizer application

The bacterial coated urea (plant growth–promoting bacteria (PGPBs)) (46% N) is the product of Jaffer Agro Services Private Limited Pakistan, and neem-coated urea (NCU) is provided by Engro Fertilizers Limited, while sulfur-coated urea (SCU) (36% N) is the product of SAFI Chemicals & Fertilizers Limited Pakistan SCU. The N was loaded on biochar by adding biochar in a solution containing dissolved simple urea N before the application. The N was applied in the prescribed treatments by using simple urea (46% N), NCU (46% N), bacterial coated urea (46% N), SCU (23% N), and loaded biochar. Half dose of N (80 kg ha^-1^, 60 kg ha^-1^, and 40 kg ha^-1^ in terms of N_1_, N_2_, and N_3_ respectively) was applied as a basal dose in all treatments. The remaining N was divided into two equal splits. The 1st split of N (40 kg ha^-1^, 30 kg ha^-1^, and 20 kg ha^-1^ in terms of N_100_, N_75_, and N_50_ respectively) was applied with the 2nd irrigation. The 2nd split dose of N (40 kg ha^-1^, 30 kg ha^-1^, and 20 kg ha^-1^ was applied with the 4th irrigation. Recommended doses of phosphorus (90 kg ha^-1^) and potassium (60 kg ha^-1^) were applied at the time of sowing uniformly in all treatments.

#### Crop management practices

Cotton variety MNH-1020 characterized as high yielding, fertilizers responsive (Cotton Central Research Institute, Multan), was used as it is a well adapted to farmer’s field in the region. Sowing was done manually by using seed at 15 kg ha^-1^ on April 25, 2019, at a distance of 75 cm bed to bed followed by 30-cmplant-to-plant distance after opening a bed with duck foot tines and placing seeds to a depth of 4 cm and covering with soil. Before sowing, seeds were treated with mancozeb at the rate of 3 g kg^-1^ of cottonseed to prevent seed-borne diseases. In total, 16 irrigations (720 mm water) were applied to the cotton crop. Out of which, first four irrigations were applied at 4-day intervals, and 12 irrigations were applied at 12–15-day intervals after assessing the crop requirements and environment. Weeds in the plots were controlled with the pre-emergence application of pendimethalin 30% EC at 0.75 kg a.i. ha^–1^ at 2 days after sowing (DAS) followed by one hand weeding at 40 DAS.

### Measurements and analytical procedures

#### Physiological attributes

Stomatal conductance, net leaf photosynthesis rate, and net leaf transpiration rate were estimated using CIRAS from ten randomly selected tagged plants in each experimental unit at the full canopy development stage (120 days after sowing).

#### Growth, morphological, and yield attributes

Leaf area index was determined at the full canopy development stage (120 days after sowing) using the method of Sestak et al. (1971) which was recorded as peak leaf area index. Similarly, the crop growth rate was determined at the full canopy development stage (120 days after sowing) by employing the procedure of Watson (1952). Randomly ten selected plants from each experimental unit/plot were used to measure plant height from base to tip of the plant’s main stem with measuring tape. At maturity, the plants were harvested from an area of 1 m^2^ to compute the total dry matter production. The samples of the harvested plants were separated into leaves, stem, and reproductive parts and ovendried at 65–70 °C to a constant weight. The recorded dry weight of samples was converted into biological yield (kg ha^-1^). Seed cotton yield was obtained from the net plot area, was added into the seed cotton weight of already picked 10 bolls, and was presented as seed cotton yield (kg ha^-1^).

#### Determination of N use efficiency

The N use efficiency was determined by the partial factor productivity and partial nutrient balance in this experiment. Partial factor productivity (PFP) was measured by dividing the seed cotton yield (kg) per kg of N applied (Ghafoor et al. 2021).

1 $$ \mathrm{PFP}\ \left(\mathrm{kg}\ \mathrm{seedcotton}\ \mathrm{yield}\ \mathrm{per}\ \mathrm{kg}\ \mathrm{N}\ \mathrm{applied}\right)=\frac{\mathrm{Seed}\ \mathrm{cotton}\ \mathrm{yield}\ \left(\mathrm{kg}\right)}{\mathrm{N}\ \mathrm{applied}\ \left(\mathrm{kg}\right)} $$

#### Fiber quality attributes

For the determination of ginning outturn (GOT), a representative tester of 100 grams from all treatments was taken, and ginning percentage was found by using the following formula (Saleem et al., 2010, b).

2 $$ \mathrm{GOT}\ \left(\%\right)=\frac{\mathrm{Weight}\ \mathrm{of}\ \mathrm{lint}\ \left(\mathrm{g}\right)}{\mathrm{Weight}\ \mathrm{of}\ \mathrm{seed}\ \mathrm{cotton}\ \left(\mathrm{g}\right)}\times 100 $$

The fiber length and fiber strength were measured by placing a sample of lint of 2.0 grams in a computerized technology known as high volume instrument (HVI) available in the Fiber Technology Section, Central Cotton Research Institute, Multan, Pakistan.

#### Determination of soil available N

According to experimental treatments, the soil samples were collected with a soil auger from each plot and were analyzed after the application of 1st, 2nd, and 3rd dose of N. Collected soil samples from each plot from 0 to 15, 15 to 30, 30 to 60, and 60 to 90 depths (cm) were sieved (2-mm mesh) after air drying. Standard protocol and procedure of alkaline permanganate (SubbaiahV and Asija, 1956) was used for the determination of soil available N.

#### Growing degree day (°C days) estimation

Daily maximum air temperature (Tmax) and minimum temperature (Tmin) were used to compute thermal time (growing degree days) requirements above a threshold temperature (TT) in terms of degrees days (DD). Thermal time was calculated with the formula equation that calculates DD as the difference between the daily mean temperature and the threshold temperature (TT) specific for cotton crop.

3 $$ \mathrm{DD}\ \left({}^{\circ}\mathrm{C}\ \mathrm{days}\right)={\sum}_{\mathrm{i}=\mathrm{dh}}^{\mathrm{i}=\mathrm{ds}}\left[\left\{\frac{\mathrm{Tmax}+\mathrm{Tmin}}{2}\right\}-\mathrm{TT}\right] $$

where DD (°C days) accretion accumulation is the accumulative degrees days for specific pheno-phase, “ds” is the date of sowing, “dh” is the date of harvest, TT is the threshold temperature which was considered as 15 °C for cotton crop to compute the thermal time (Xue et al. 2008). In this case, if [(Tmax + Tmin)/2] < TT, or [(Tmax + Tmin)/2] = TT, then DD was considered equal to zero.

#### Statistical analysis

The recorded data (cotton growth, physiology, morphological, yield and quality attributes, and the N use efficiency of a cotton crop) was analyzed statistically using analysis of variance (ANOVA) to assess the effects of biochar and slow-release nitrogenous fertilizers on cotton growth, physiology, morphology, yield, and quality attributes and the NUE using SAS version 9.4 (SAS Institute, Cary, NC 2013). The general linear mixed model (GLM) was used while the effects of each treatment were assessed separately and collectively for all studied parameters. Moreover, the mean separation test (Tukey’s honest significant difference (HSD)) was engaged to separate differences between the treatment means and was considered significant at *p ≤ 0.05.* The correlation and regression analysis were done for obtained clear results between assorted treatments (Steel et al. 1997). Furthermore, data were also analyzed using Sigma Plot 11.0 (Systat, Inc., Richmond, USA) at a significance level of *p ≤ 0.05*.

## Results

### Physiological attributes

**Figure 3:**
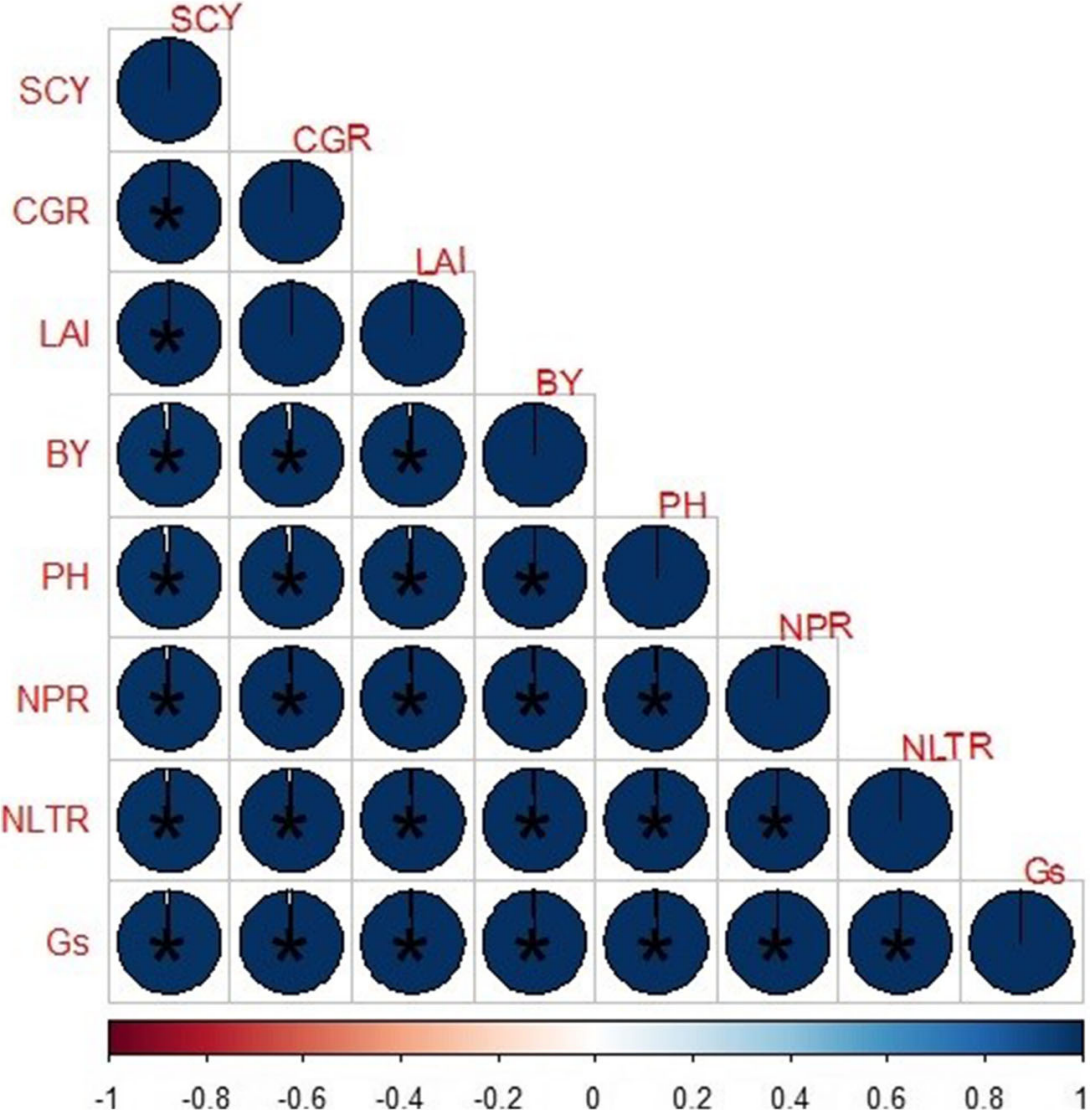
Correlation of different parameters of cotton growth, yield and physiological attributes under different slow-release N fertilizers and N increments. The areas of circles show the absolute value of corresponding correlation coefficients tested at *0.01 significance level. SCY, cottonseed yield; PH, plant height; LAI, peak leaf area index; BY, biological yield; NLTR, net leaf transpiration rate; Gs, stomatal conductance; NPR, net photosynthetic rate. *, p ≤ 0.05; **, ≤ 0.01; ***, ≤ 0.001

The effects of different slow-release N fertilizers and N increments were significant for the physiological attributes (net leaf photosynthetic rate, net leaf transpiration rate, and stomatal conductance) at *p* ≤ 0.05. Similarly, two-way interactions were also significant at *p* ≤ 0.05 for all studied physiological attributes (Table 2). Cotton fertilized with bacterial coated urea at higher N application (160 kg ha^-1^) showed a higher net leaf photosynthetic rate (32.8 μmol m^-2^ s^-1^) and leaf stomatal conductance (0.502 mol m^-2^ s^-1^) in comparison to simple urea application (160 kg ha^-1^), respectively. Similarly, cotton crop fertilized with bacterial coated urea at higher N application (160 kg ha^-1^) showed a higher net leaf transpiration rate (10.8 mmol s^-1^) in comparison to simple urea application (160 kg ha^-1^), respectively (Table 2). Net leaf photosynthetic rate, net leaf transpiration rate, and stomatal conductance showed a significant positive Pearson correlation with each other (Figure 3).

**Table 2:** Effect of different slow-release N fertilizers and N increments on physiological attributes of cotton crop

**Treatments**	**Net leaf photosynthesis rate (μmol m**^**-2**^**s**^**-1**^**)**	**Stomatal conductance** **(mol m**^**-2**^**s**^**-1**^**)**	**Net Leaf** **transpiration rate** **(mmol s**^**-1**^**)**
**Simple urea**			
*N*_1_=160 kg ha^-1^	31.2 b	0.478 b	10.3 b
*N*_2_= 120 kg ha^-1^	25.6 cd	0.392 cd	8.44 cd
*N*_3_= 80 kg ha^-1^	20.8 h	0.317 h	6.81 h
**Neem-coated urea**			
N_1_=160 kg ha^-1^	31.3 b	0.479 b	10.3 b
N_2_= 120 kg ha^-1^	26.4 c	0.403 c	8.67 c
N_3_= 80 kg ha^-1^	22.2 g	0.339 g	7.30 g
**Sulfur-coated urea**			
N_1_=160 kg ha^-1^	31.2 b	0.478 b	10.3 b
N_2_= 120 kg ha^-1^	24.3 e	0.371 e	8.00 de
N_3_= 80 kg ha^-1^	20.7 h	0.316 h	6.80 h
**Bacterial coated urea**			
N_1_=160 kg ha^-1^	32.8 a	0.502 a	10.8 a
N_2_= 120 kg ha^-1^	31.4 b	0.485 b	10.5 b
N_3_= 80 kg ha^-1^	23.2 f	0.355 f	7.63 f
**Loaded biochar**			
N_1_=160 kg ha^-1^	31.2 b	0.479 b	10.3 b
N_2_= 120 kg ha^-1^	24.7 de	0.379 de	8.15 e
N_3_= 80 kg ha^-1^	20.9 g	0.319 h	6.87 h
SRF	**	**	**
NI	**	**	**
SRF × NI	**	**	**

N_1_, 100% of recommended N; N_2_, 75% of recommended N; N_3_, 50% of recommended N; SRF, slow-release N fertilizers; NI, N increments; **, significant at *p*≤0.01

### Growth, morphological, and yield attributes

The effects of different slow-release N fertilizers and N increments were significant for the morphological and yield attributes (plant height, peak leaf area index, boll weight, seed cotton yield, and biological yield) and N use efficiency at *p* ≤ 0.05. And the two-way interactions NN were also significant at *p* ≤ 0.05 for all studied morphological and yield attributes and N use efficiency (Table 3). Cotton fertilized with bacterial coated urea at higher N application (160 kg ha^-1^) showed higher plant height (150 cm) in comparison to simple, neem, sulfur, and biochar-loaded urea application (160 kg ha^-1^), respectively (Table 3). Moreover, cotton fertilized with different material coated urea showed nonsignificant results at higher N application (160 kg ha^-1^), while the maximum peak leaf area index (3.90) showed by bacterial coated urea in comparison to other coatings of urea fertilizers (Table 3). Similar results were found in the case of observing cotton crop growth rate by application of coated urea under arid environmental conditions. Moreover, cotton fertilized with bacterial coated urea at higher N application (160 kg ha^-1^) showed a maximum 8.55% increase in seed cotton yield (4662 kg ha^-1^) as compared to simple urea, but other coating materials of urea showed statistically similar results at the same N level (Table 3). Different coating materials of urea were not shown a significant increase in biological yield under field conditions. Cotton fertilized with bacterial coated urea at lower N application (80 kg ha^-1^) showed higher N use efficiency (39.6 kg SCY/N kg) in comparison to 120 kg ha^-1^and 160 kg ha^-1^(Table 3). However, cotton crop fertilized with NCU, SCU, and loaded biochar at higher N application (160 kg ha^-1^) showed similar plant height, peak leaf area index, mean boll weight, and seed cotton yield recorded with the application of simple urea application (160 kg ha^-1^) (Table 3). Moreover, cotton showed a higher peak leaf area index, peak crop growth rate, and seed cotton yield with the application of bacterial coated urea at 120 kg ha^-1^ N application rate (Table 3). Plant height, peak leaf area index, boll weight, seed cotton yield, and biological yield showed a significant positive association with each other (Figure 3).

**Table 3:** Effect of different slow-release N fertilizers and N increments on morphological and yield attributes of a cotton crop

**Treatments**	**Plant height (cm)**	**Leaf area index (LAI)**	**CGR** **g m**^**2**^**day**^**-1**^**)**	**Seed cotton** **yield (kg ha**^**-1**^**)**	**Biological yield (kg ha**^**-1**^**)**	**N use efficiency (kg SCY/kg N)**
**Simple urea**						
N_1_=160 kg ha^-1^	143 b	3.73 a	3.96 a	4254 b	10107 b	26.6 f
N_2_= 120 kg ha^-1^	117 cd	3.04 c	3.24 c	3521 d	8292 cd	29.3 e
N_3_= 80 kg ha^-1^	103 g	2.43 g	2.62 f	2823 e	7126 g	35.3 c
**Neem-coated urea**						
N_1_=160 kg ha^-1^	143 b	3.73 a	3.94 a	4247 b	10133 b	26.5 f
N_2_= 120 kg ha^-1^	120 c	3.29 b	3.56 b	3827 c	8517 c	31.9 d
N_3_= 80 kg ha^-1^	102 fg	2.67 ef	2.80 ef	3017 e	7195 fg	37.7 ab
**Sulfur-coated urea**						
N_1_=160 kg ha^-1^	143 b	3.75 a	3.95 a	4246 b	10109 b	26.5 f
N_2_= 120 kg ha^-1^	111 e	2.91 cd	3.07 cd	3307 d	7854 e	27.6 ef
N_3_= 80 kg ha^-1^	99 g	2.48 g	2.61 f	2810 e	7035 g	35.1 c
**Bacterial coated urea**						
N_1_=160 kg ha^-1^	150 a	3.90 a	4.15 a	4652 a	10613 a	27.9 ef
N_2_= 120 kg ha^-1^	143 b	3.77 a	4.01 a	4513 a	10261 b	35.9 bc
N_3_= 80 kg ha^-1^	106 f	2.78 de	2.94 de	3369 d	7487 f	39.6 a
**Loaded biochar**						
N_1_=160 kg ha^-1^	143 b	3.74 a	3.94 a	4254 b	10114 b	26.6 f
N_2_= 120 kg ha^-1^	113 de	2.97 c	3.13 cd	3366 d	8004 de	28.0 ef
N_3_= 80 kg ha^-1^	101 g	2.49 fg	2.64 f	2840 e	7150 g	35.5 c
SRF	**	**	**		**	**
NI	**	**	**		**	**
SRF × NI	**	**	**		**	**

N_1_, 100% of recommended N; N_2_, 75% of recommended N; N_3_, 50% of recommended N; SRF, slow-release N fertilizers; NI, N increments; **, significant at *p*≤0.01; ; CGR, crop growth rate (g m^-2^ day^-1^); SCY, seed cotton yield (kg ha^-1^). LAI and CGR are recorded at 90 DAS while seed cotton yield, biological yield, and NUE are recorded and computed at the final picking stage

### Quality attributes

**Figure 4:**
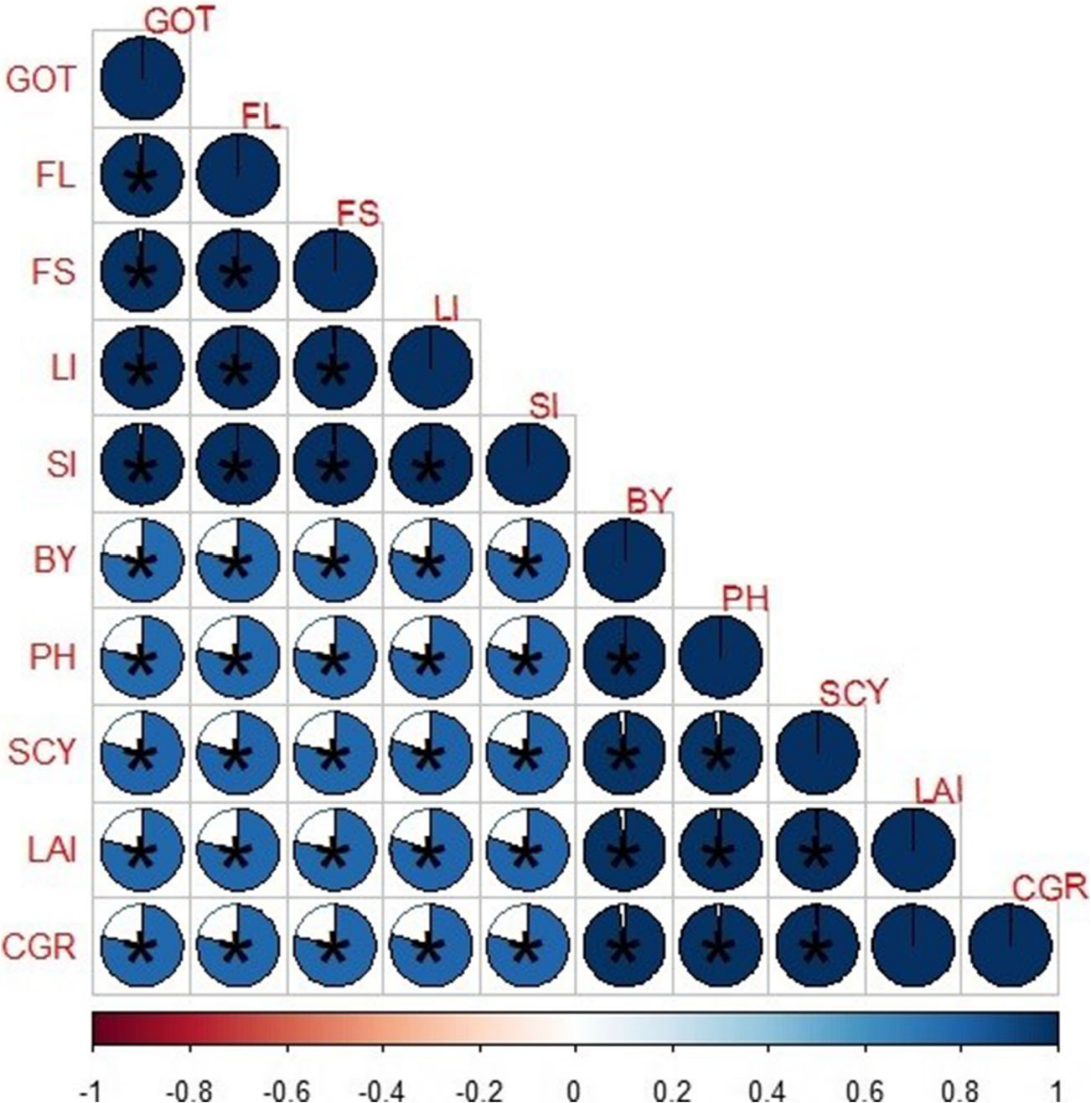
Correlation of different parameters of cotton growth, yield, and quality attributes under different slow-release N fertilizers and N increments. The areas of circles show the absolute value of corresponding correlation coefficients tested at *0.01 significance level. SCY, seed cotton yield; PH, plant height; LAI, peak leaf area index; CGR, crop growth rate; BY, biological yield; FL, fiber length; FS, fiber strength; LI, lint index; GOT, ginning outturns; SI, seed index

The effects of different N increments were significant for the quality attributes (fiber length, fiber strength, ginning outturn, fiber index, and seed index) at *p* ≤ 0.05 (Table 4). However, the effects of different slow-release N fertilizers and two-way interactions NN were nonsignificant at *p* ≤ 0.05 for all studied quality attributes (Table 4). Cotton fertilized with higher N application (160 kg ha^-1^) showed higher fiber length (27.4 cm) and fiber strength (27.0 cm) in comparison to that of N_2_ (120 kg ha^-1^) and N_3_ (80 kg ha^-1^) N application (Table 4). Similarly, cotton fertilized with a higher N application rate (160 kg ha^-1^) showed a higher ginning outturn (42.5%) in comparison to N_2_ (120 kg ha^-1^) and N_3_ (80 kg ha^-1^) N application (Table 4). Likewise, cotton fertilized with higher N application (160 kg ha^-1^) showed higher fiber index (5.62 g) and seed index (7.61 g)in comparison to N_2_ (120 kg ha^-1^) and N_3_ (80 kg ha^-1^) N application (Table 4). Fiber length, fiber strength, ginning outturn, fiber index, and seed index showed a significant positive Pearson correlation associated with each other (Figure 4).

**Table 4:** Effect of different slow-release N fertilizers and N increments on cotton quality attributes under arid environment

**Treatments**	**Fiber length (cm)**	**Fiber strength (cm)**	**Ginning out turn (%)**	**Fiber index (g)**	**Seed index (g)**
N_100_=160 kg ha^-1^	27.4 A	27.0 A	42.4 A	5.62 A	7.61 A
N_75_=120 kg ha^-1^	26.8 B	26.4 B	41.8 B	5.36 B	7.44 B
N_50_=80 kg ha^-1^	25.9 C	25.5 C	40.8 C	4.97 C	7.17 C
SRF	NS	NS	NS	NS	NS
NI	**	**	**	**	**
SRF × NI	NS	NS	NS	NS	NS

N_100_, 100% of recommended N; N_75_, 75% of recommended N; N_50_, 50% of recommended N; SRF, slow-release N fertilizers; NI, N increments; **, significant at *p*≤0.01; NS, nonsignificant at *p*≤0.05

### Soil available nitrogen

**Figure 5:**
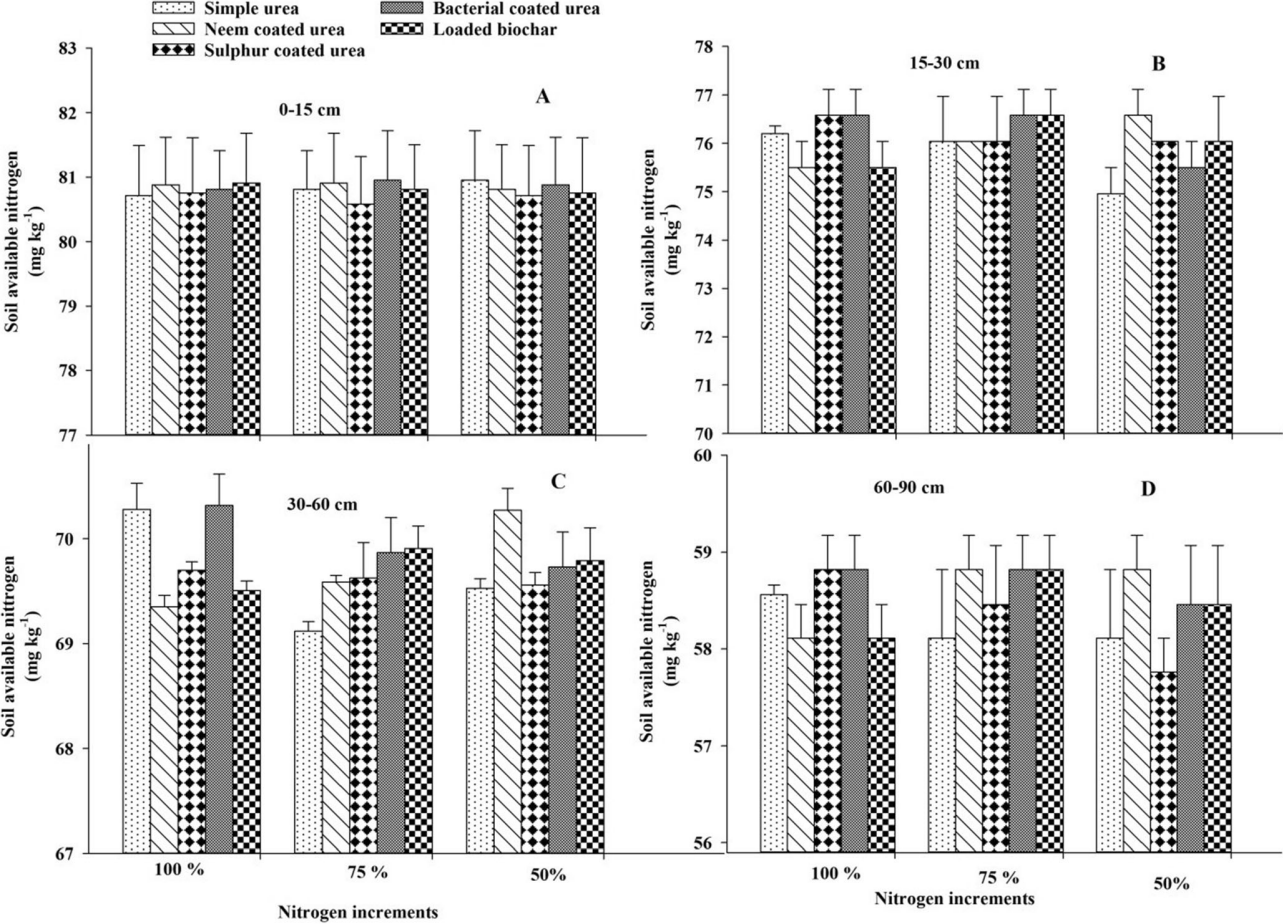
Effect of slow-release N fertilizers and increments on soil available N under arid climatic conditions at different soil depths (0–15 cm = A, 15–30 cm = B, 30–60 cm = C, and 60–90 cm = D) after 30 DAS of cotton crop. Error bar showed standard error from mean values

**Figure 6:**
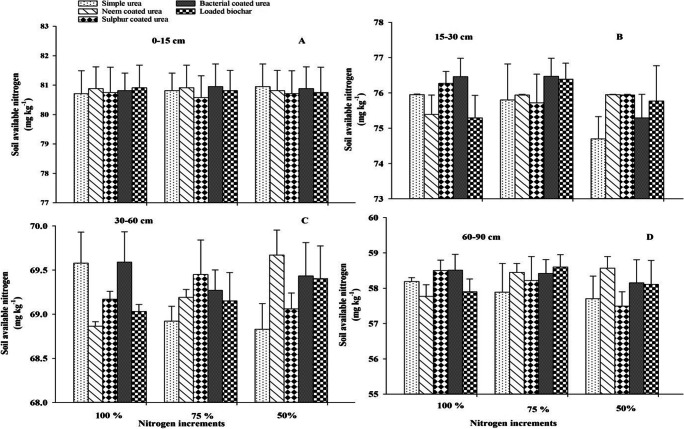
Effect of slow-release N fertilizers and increments on soil available N under arid climatic conditions at different soil depths (0–15 cm = A, 15–30 cm = B, 30–60 cm = C, and 60–90 cm = D) after 60 DAS of cotton crop. Error bar showed standard error from mean values

**Figure 7:**
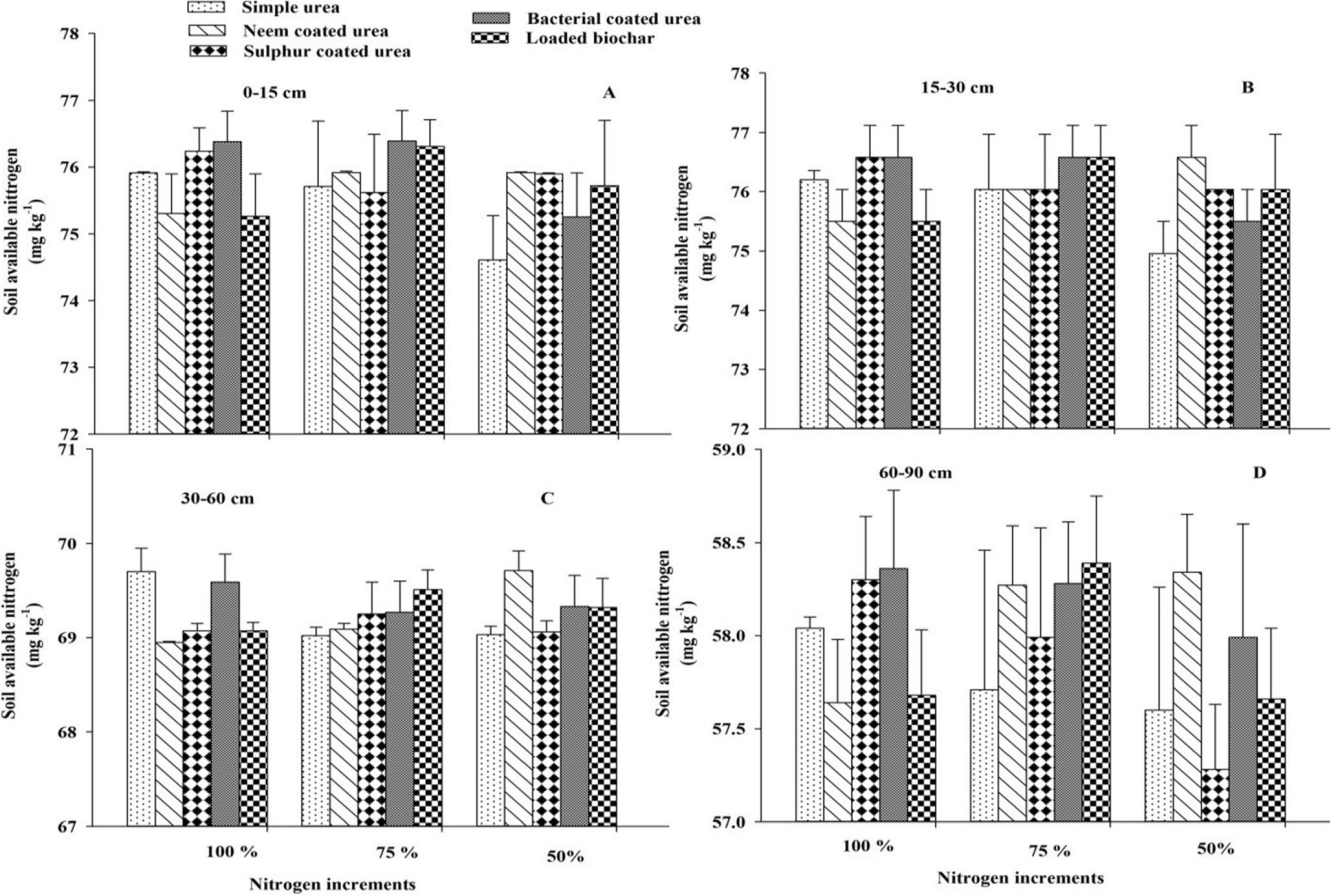
Effect of slow-release N fertilizers and increments on soil available N under arid climatic conditions at different soil depths (0–15 cm = A, 15–30 cm = B, 30–60 cm = C, and 60–90 cm = D) after 60 DAS of cotton crop. Error bar showed standard error from mean values

Soil available nitrogen did not differ due to the application of different slow-release nitrogen fertilizers and nitrogen increments at *p* ≤ 0.05. Two-way interaction was not significant at *p* ≤ 0.05 for soil available nitrogen. However, soil available nitrogen increased with increasing soil depth and difference response regarding N application and source can be seen in Figures 5, 6, and 7.

## Discussion

Cotton plants need macronutrients consistently and in higher amounts for optimum growth and development which leads to higher seed cotton yield and improved lint quality. Monotypic urea has high solubility leading to losses through denitrification, nitrate leaching, and ammonia volatilization which not only leads to reduced seed cotton yield but also causes soil degradation, greenhouse gases emission, and groundwater pollution (Spiertz, 2009; Guo et al. 2010; Kawakami et al. 2012; Zhang et al. 2012; Ghafoor et al. 2021). In the present study, we used the different slow-release N fertilizers in combination with different N increments to improve the N use efficiency and seed cotton yield in cotton production.

In the present study, plant height and peak leaf area index increased with the application of coated urea at higher N application (160 kg ha^-1^) over simple urea (160 kg ha^-1^) which might be due to sufficient availability of N from the slow release of bacterial (PGPB) coated urea (Singh et al. 2019). This slow release of N corresponded well and might substantially be supported in the fast development of roots and leaves due to more production of chlorophyll content resulting in higher biomass accumulation and ultimately higher plant height and peak LAI (Silvertooth et al. 2011; Geng et al. 2016). Moreover, improved plant height and peak LAI might be because of maximum canopy development which resulted in maximum net photosynthetic rate and assimilate portioning and ultimately higher plant height and peak leaf area index (Wang, 2013; Tian et al. 2017). Several studies have reported that plant height and LAI increase with the application of slow-release N fertilizers in cotton production (Li et al. 2007; Geng et al. 2016). Furthermore, previous research has also reported that sunflower showed the highest plant height, total dry matter with the application of polymer-coated urea as compared with common urea (Pareveen et al. 2021).

In the current study, physiological attributes, i.e., net leaf photosynthetic rate, stomatal conductance, and net leaf transpiration rate were increased with the application of bacterial coated urea at higher N application (160 kg ha^-1^) over simple urea application (160 kg ha^-1^). This might be because of enhanced N availability and maximum synthesis of chlorophyll content which leads to higher light capturing and ultimately enhanced net leaf photosynthesis rate (Silvertooth et al. 2011; Singh et al. 2010). Furthermore, a higher net leaf photosynthesis rate increased the canopy development which resulted in a greater number of stomata required for higher stomatal conductance and net leaf transpiration rate. Likewise, higher net leaf photosynthetic rate, chlorophyll content, and stomatal conductance of a sunflower crop were recorded with the application of polymer-coated urea in comparison to common urea under an arid environment (Perveen et al., 2021). The leaf spade value, soil inorganic N contents, and net photosynthetic rate were enhanced by using controlled release fertilizers especially from the full bloom phase to the boll opening phase of cotton (Tian et al. 2017).) In the present study, seed cotton yield (SCY) showed little increase with the application of bacterial-coated urea at higher N application over simple urea application (160 kg ha^-1^) which might be due to the increasing trend in total bolls per plant and mean boll weight which are directly related with seed cotton yield. Similar results were also found by Geng et al. (2016) who reported that seed cotton yield was higher in slow-release N fertilizer (polymer-coated urea) at higher N application in comparison to simple urea application. Furthermore, sulfur, neem, and biochar loaded urea showed nonsignificant results of biological yield, but a little increase observed with the application of bacterial coated urea at high N level (160 kg ha^-1^) over other sources of urea application might be due to increasing trend in plant height, number of monopodial, and sympodial branches which are directly related with biological yield. Similar results were also found by Geng et al. (2016) which reported that seed cotton yield was higher in slow-release N fertilizer (polymer-coated urea) higher N application than simple urea application. The N use efficiency increased with the application of bacterial coated urea at lower N application which might be because of slow release of N from bacterial coated urea and reduced N losses through leaching, volatilization, and denitrification (Tang et al. 2012; Khan et al. 2018, 2021). Geng et al. (2016) also found that the N use efficiency of a cotton crop was higher in slow-release N fertilizer (polymer-coated urea) in comparison to simple urea application. Furthermore, Perveen et al. (2021) also found that the N use efficiency of a sunflower crop was higher with the application of polymer-coated urea as compared with common urea.

Quality attributes are always the result of the interactive effects of genotype, cultivar, weather attributes, soil conditions, and crop management practices (Scharf and Lory, 2002; Dong et al. 2006). Among crop management practices, fertilizer management (N fertilization) is the main factor that affects the quality attributes of cotton. Similarly, several investigations have proved that deficiency of N decreased the quality attributes especially fiber length and fiber strength (Scharf and Lory, 2002; Dong et al. 2006; Read et al. 2006). In the present study, fiber length, fiber strength, ginning outturn, fiber index, and seed index were improved when cotton was fertilized with higher N application (160 kg ha^-1^) in comparison to the second (120 kg ha^-1^) and third (80 kg ha^-1^) N application rate, respectively, which was attributed to the adequate N supply during the whole crop period. Several studies have presented improvements in the quality attributes of cotton with the application of higher N application in comparison to lower N application (Hallikeri et al., 2010; Saleem et al., 2010, b).

## Conclusions

In the present study, we showed that different slow-release N fertilizers and biochar in combination with different N increments have markedly affected growth, morphological, physiological, and yield attributes and the N use efficiency of a cotton crop. Similarly, different N increments have affected the quality attributes of cotton crop. The highest growth and morphological, physiological, and yield attributes of a cotton crop were obtained when it was fertilized with bacterial coated urea at higher N application (160 kg ha^-1^). However, bacterial coated urea with lower N application (80 kg ha^-1^) showed the highest N use efficiency. Furthermore, the cotton crop showed improved quality attributes with higher N application. In short, bacterial coated urea with recommended (160 kg ha^-1^) and 75% of recommended N application (120 kg ha^-1^) may be recommended to enhance the seed cotton yield and the NUE in the cotton production system of the region. Future research suggested including the process-based mechanistic modeling approaches to understand the basic process of N dynamics in different soil layers and to monitor the N losses for different slow-release and biochar loaded N fertilizers.
